# Neutrophil gelatinase-associated lipocalin predicts postoperative fluid overload, a potentially modifiable risk factor for mortality after cardiac surgery

**DOI:** 10.1186/cc10957

**Published:** 2012-03-20

**Authors:** M Haase, P Mertens, M Plaß, R Bellomo, A Haase-Fielitz

**Affiliations:** 1 Otto-von-Guericke University Magdeburg, Germany; 2 German Heart Center, Berlin, Germany; 3 Austin Health, Melbourne, Australia

## Introduction

In most previous studies, neutrophil gelatinase-associated lipocalin (NGAL), measured immediately following cardiac surgery, has been demonstrated to predict postoperative increases in serum creatinine and decline in urine output. In patients undergoing cardiac surgery, postoperative fluid overload is a typical complication. In this study, we investigated the early postoperative value of NGAL to predict subsequent fluid overload, a potentially modifiable risk factor in these patients.

## Methods

We studied 100 adult cardiac surgery patients assigned to the control arm of a randomized controlled trial. Urine and serum were sampled immediately after admission to the ICU. Urine NGAL was measured on the ARCHITECT laboratory platform (Abbott Diagnostics) and serum creatinine using an enzymatic assay. Postoperative fluid overload was defined as positive fluid balance with >10% excess of preoperative body weight within 48 hours. An area under the curve of the receiver-operating characteristics (AUC-ROC) of 0.5 indicates the predictive ability equaling the toss of a coin and >0.7 of a useful biomarker.

## Results

Postoperative fluid overload was present in 15% of patients with a mean positive fluid balance of 12 ± 9 kg. Patients who survived the hospital stay had a lower positive fluid balance of 2.8 l (25th to 75th percentiles: 1.5 to 5.5) compared to patients who died (23.0 l (25th to 75th percentiles: 14.5 to 24.0)); *P *= 0.010. A positive fluid balance predicted in-hospital mortality with AUC 0.94 (95% CI 0.83 to 0.99), sensitivity 100% and specificity 80% at a cut-off >6 l. Urine NGAL predicted fluid overload (AUC-ROC 0.80 (95% CI 0.64 to 0.93)) and mortality (AUC-ROC 0.88 (95% CI 0.78 to 0.97)). Serum creatinine did not predict fluid overload (AUC-ROC 0.51 (95% CI 0.24 to 0.78)) or mortality (AUC-ROC 0.61 (95% CI 0.16 to 0.99)).

## Conclusion

Fluid overload frequently occurs during the first 48 hours after cardiac surgery and is strongly correlated with postoperative mortality. Early postoperatively measured urine NGAL is a good predictor of fluid overload and mortality whereas measurement of serum creatinine at the same time equals the toss of a coin. Early NGAL-guided adjustments of fluid management might reduce organ edema and potentially improve patient outcomes after cardiac surgery. Our findings should be validated in larger patient cohorts.

